# Flawed Ethics Recommendations of the U.S. EPA’s Human Studies Review Board

**DOI:** 10.1289/ehp.115-a17

**Published:** 2007-01

**Authors:** Kristin Shrader-Frechette

**Affiliations:** Department of Biological Sciences, Department of Philosophy, University of Notre Dame, Notre Dame, Indiana, E-mail: kshrader@nd.edu

The U.S. Environmental Protection Agency’s (EPA) new rule to protect human research subjects has generated scientific, ethical, and legal controversy (Burton 2006). Addressing pesticide studies submitted by third parties to the U.S. EPA for possible use in regulatory decisions, the rule also authorized an independent Human Subjects Review Board (HSRB) to evaluate these studies. How successful has the HSRB been?

The board’s first report (HSRB 2006), a scientific and ethical review of third-party, intentional human-exposure studies on eight active ingredients used in pesticides, was issued 26 June 2006. The HSRB (2006) concluded that studies of seven pesticides [aldicarb, amitraz, azinphos-methyl, dichlorvos (DDVP), ethephon, methomyl, and oxamyl] “failed to fully meet the specific ethical standards prevalent at the time the research was conducted …” (see also Lockwood 2004; Needleman et al. 2005; Oleskey et al. 2004; Sass and Needleman 2004). Nevertheless, the HSRB (2006) concluded that

There was no clear and convincing evidence that the research [on these seven pesticides] was fundamentally unethical—intended to seriously harm participants or that informed consent was not obtained.

This second HSRB conclusion is ethically questionable on several grounds. First, it relies on an arbitrary definition of “fundamentally unethical” research as either intended to seriously harm participants or that fails to obtain informed consent. Yet neither the U.S. EPA (2006) nor the National Research Council (NRC 2004) defines “fundamentally unethical” so narrowly. Instead, both say only that studies which intend harm or violate consent are examples of “fundamentally unethical” research.

In reducing “fundamentally unethical” research to only two types of problems, the HSRB excludes much behavior that ethicists traditionally have condemned. Negligence and culpable ignorance (Aristotle 1985)—as well as lying, using people as means to an end, or pursuing self-interest at the expense of others (Kant 1964)—are unethical, even without intent to harm others.

To assume that bad intentions are required to make serious harms fundamentally unethical also ignores “errors of omission” and focuses merely on commission—having harmful intent. Yet researchers err through omission if they behave irresponsibly toward their subjects: Perhaps they intend no harm, but through laziness, greed, or carelessness (Aristotle 1985), they fail to recognize subjects’ manifesting harmful symptoms.

The second HSRB conclusion also imposes an unfair burden on research victims or opponents, requiring them to establish researchers’ intentions. Yet intentions are almost impossible to know; they are private—not empirical—and thus typically known only by the individual. Proof of intent to harm is not required to judge bank robbers or white-collar criminals. Why should evaluators of research have such an unfair burden?

One reason for the HSRB’s questionable ethical conclusions may be inadequate bioethics expertise. No board members have terminal degrees in bioethics or even ethics. Fields represented are anesthesiology, environmental health sciences (2), epidemiology, medicine, microbiology, neurology, pharmacology (3), psychology, statistics (2), and toxicology (3) (HSRB 2006). The U.S. EPA also has not followed recommendtions of its Science Advisory Board (2000), the NRC (2004), and the Environmental Medicine Workgroup (Oleskey et al. 2004) to establish specific ethics guidelines for all U.S. EPA-related research. Without such guidelines (e.g., avoid low-power studies), questionable ethical conclusions likely will continue.
